# Including Health in Environmental Assessments of Major Transport Infrastructure Projects: A Documentary Analysis

**DOI:** 10.15171/ijhpm.2017.55

**Published:** 2017-05-10

**Authors:** Emily Riley, Patrick Harris, Jennifer Kent, Peter Sainsbury, Anna Lane, Fran Baum

**Affiliations:** ^1^Menzies Centre for Health Policy, Sydney, NSW, Australia.; ^2^School of Public Health, and Sydney Medical School, The University of Sydney, Sydney, NSW, Australia.; ^3^Urban and Regional Planning, Faculty of Architecture, Design, and Planning, The University of Sydney, Sydney, NSW, Australia.; ^4^Population Health, South Western Sydney Local Health District, Sydney, NSW, Australia.; ^5^Southgate Institute for Health, Society, and Equity, Flinders University, Bedford Park, SA, Australia.

**Keywords:** Health, Transport, Infrastructure, Environmental Assessment (EA), Content Analysis

## Abstract

**Background:** Transport policy and practice impacts health. Environmental Impact Assessments (EIAs) are regulated public policy mechanisms that can be used to consider the health impacts of major transport projects before they are approved. The way health is considered in these environmental assessments (EAs) is not well known. This research asked: How and to what extent was human health considered in EAs of four major transport projects in Australia.

**Methods:** We developed a comprehensive coding framework to analyse the Environmental Impact Statements (EISs) of four transport infrastructure projects: three road and one light rail. The coding framework was designed to capture how health was directly and indirectly included.

**Results:** We found that health was partially considered in all four EISs. In the three New South Wales (NSW) projects, but not the one South Australian project, this was influenced by the requirements issued to proponents by the government which directed the content of the EIS. Health was assessed using human health risk assessment (HHRA). We found this to be narrow in focus and revealed a need for a broader social determinants of health approach, using multiple methods. The road assessments emphasised air quality and noise risks, concluding these were minimal or predicted to improve. The South Australian project was the only road project not to include health data explicitly. The light rail EIS considered the health benefits of the project whereas the others focused on risk. Only one project considered mental health, although in less detail than air quality or noise.

**Conclusion:** Our findings suggest EIAs lag behind the known evidence linking transport infrastructure to health. If health is to be comprehensively included, a more complete model of health is required, as well as a shift away from health risk assessment as the main method used. This needs to be mandatory for all significant developments. We also found that considering health only at the EIA stage may be a significant limitation, and there is a need for health issues to be considered when earlier, fundamental decisions about the project are being made.

## Background

Transport networks and practices are well recognised determinants of health. Transport policy and planning, particularly in urban settings, affect health in various ways.^1,2^ Transport investment decisions influence the availability of different modes of transport (for example cars, public transport, walking and cycling) and subsequently travel behaviour. This exposes populations to air and noise pollution, traffic accidents and personal danger, physical (in)activity, and opportunities for social engagement. Such exposure may influence health outcomes including but not limited to cardiovascualar and respiratory conditions, injuries, obesity and mental illness. Each of these impacts on quality of life, and increases health care needs and costs. These health impacts are influenced by population characteristics including demographics and socio-economic status. The recommendations from this now large body of evidence revolve around better integration of transport and urban planning that reduces reliance on carbon dependent private cars and enhances opportunities for walking, cycling and public transport.^3^

The importance of engaging with health issues in urban sustainability decision making (including transport) has been highlighted in recent global statements such as Habitat 21,^4^ and improving both health and urban infrastructure are now cemented as sustainable development goals.^5^ From a policy perspective, however, health oriented research and practice has yet to engage fully with the procedures that underpin transport planning decisions. One such policy mechanism is environmental impact assessment (EIA), variations of which are used within 190 of 193 United Nations (UN) member states.^6,7^ The aim of an EIA is to support decision-makers by providing a systematic analysis of the possible environmental effects of an anticipated project.^8,9^ This is done mostly in the main output of an EIA, an environmental impact statement (EIS). Recognised globally as a significant aid to decision making,^10^ best practice EIA allows the public, proponents and regulators to understand and discuss the potential impacts of a project, as well as shape its construction and operation.

A principal challenge in research, policy and practice relating to EIAs is the way health issues are framed. Previous analyses of EISs in a range of project types, not specific to transport, have shown broad consistency concerning issues that are included and excluded.^11,12^ Environmental health concerns associated with changes to the bio-physical environment such as air, soil and water quality, have long been considered in EIA, including transport projects.^13,14^ These approaches tend to favour the identification and quantification of toxicological hazards in the environment to which populations will be exposed.^14-18^ The relationship between health outcomes of people and changes to the bio-physical environment is implied, but seldom explicitly incorporated or calculated.^12,14,19^ The wider influences on health, for example those associated with socio-economic factors, or the ‘social determinants of health,’ are rarely included.^14,20-22^ This is despite global shifts which are broadening the issues considered in EIAs^9^ and a growing awareness that a focus on assessing specific risks known to health may miss many uncertainties and ambiguities associated with a project that may threaten people’s health.^23^

In Australia, the setting for this research, the EIA process is called environmental assessment (EA). In most jurisdictions EAs are triggered when projects reach a certain monetary cost or are judged to have a potentially significant impact. While triggers and requirements for EA differ between jurisdictions its history is one of strong input from environmental health, and a consistent focus on health risk assessment as the method to do this,^12,24^ As with other countries, there is policy recognition in Australia of the value of investing in transport projects as core drivers of economic and social outcomes.^25^ There is, however, a lack of recent empirical investigation into the quality of health’s inclusion in transport focussed EAs as part of the planning for these projects. While the focus on risks may be necessary the known evidence about the linkages between transport and health suggest that this focus may be insufficient. For policy-makers, in health and transport infrastructure sectors in Australia and internationally, unpacking how, and the extent to which, health is framed in current EIA practice, will allow for a more critical engagement with EIA as a regulated policy procedure.

The aim of this study is to determine the degree to which and how health issues are included in EIA for large scale transport infrastructure projects. We examined the EA process of four major transport infrastructure projects in two Australian states; three in New South Wales (NSW) and one in South Australia (SA) (Table 1). Specifically, the research addresses the research question:

**Table 1:** Parameters of the 4 Transport Infrastructure Projects That Were the Focus of This Research

	**NorthConnex**	**CSELR**	**WestConnex M4 East**	**2010 Darlington Upgrade**
Project type	Road	Light Rail	Road	Road
Project description	9 km-long tolled motorway tunnel located in north Sydney	12 km-long light rail project in central Sydney	Motorway scheme, including a tunnel, in central Sydney	An upgrade to a road intersection in the Darlington area of Adelaide
Jurisdiction	NSW	NSW	NSW	SA
Cost (Au$)	3 billion	1.6 billion	16.8 billion	630 million
Funded by	A public private partnership between the Australian Government, NSW Government and Transurban	A public private partnership between the NSW Government and the Connecting Sydney consortium	Australian Government and NSW Government	Australian Government and South Australian Government
Commencement date	2015	2015	2016	2014
Expected completion date	2019	2019	2023	At the time of writing this project is undergoing a process of redesign and expected completion date has not been publicised

Abbreviations: NSW, New South Wales; SA, South Australia.

‘*How and to what extent is human health considered in EAs of major transport infrastructure projects?*’

To answer this question, we developed a documentary analysis coding framework to enable greater scrutiny of the quality of health coverage in EISs.

Our overall objective is to help foster greater understanding of the functioning of EIA systems to help public health practitioners engage in public policy discussions about transport infrastructure projects. The analysis also shows how, using our approach and framework, policy-makers and affected communities can assess the quality of the content of EISs from a comprehensive public health impact perspective.

## Methods

This research is part of a larger research program using established social science methods to understand how health issues are considered in urban and regional planning.^26^ The research reported here focuses on one aspect of that system with one method: EA of major infrastructure projects through documentary analysis of EISs. Further insight into the EA process is being developed concurrently from interviews with stakeholders.

### Multiple Case Design and Inclusion Criteria

We applied a multiple explanatory case study design.^27^ Multiple explanatory case studies focus on how and why phenomena occur, where each case demonstrates or uncovers specific findings which are then either replicated, or not, in other cases.^27^ Each case was developed and then compared for replication purposes in terms of similarities and differences. Therefore, we included cases with similar and different contextual conditions using the following inclusion criteria:

The project being assessed has major transport planning implications and has been declared a ‘state significant’ project by government.

• The project’s planning is at a stage where the EIS or equivalent has been produced and is publicly available.

The project is likely to have health and wellbeing considerations within the local community and/or regionally (ie, were planned to be constructed in existing urban areas).

The project is located in same state/territory as the researchers given the limited resources available to study.

### Analytic Validity Focussed Sampling

Using publicly available registers of EISs, a sample of projects was identified following the multiple case study approach advocated by Yin^27^ where the purpose is analytic validity as opposed to sampling validity (ie, the purpose of the cases was to test and replicate our analytic framework not to generate statistically comparable experimental data). The initial case and entry point, NorthConnex, was selected following a review of the publicly available EISs from the Government’s database of major projects (http://majorprojects.planning.nsw.gov.au/). NorthConnex was chosen as the first case to develop given this was a state significant road project, a tunnel, in an urban area with health included in the original requirements. Following this, we selected WestConnex M4 East as a similar case of a tunnel in an urban area with health included in the requirements for the EIA, to replicate our analytical framework. We then selected the CBD and South East light rail project as a divergent case (a light rail project without health being included in the EIA requirements) to further apply the framework on a different type of project. We then selected the Darlington project as a similar project (a road in an urban area), but within a different planning jurisdiction and context against which to reapply the analytical framework.

Brief details of the projects are provided in Table 1. In Sydney, Australia’s largest city, we selected three EISs. NorthConnex and WestConnex M4 East are roads projects; ‘health’ was included in the requirements that are issued to the proponent by the NSW Department of Planning and Environment. These requirements establish the content and issues that are to be covered in the EIS. The third Sydney project, the CSELR, is a light rail project and did not officially require health to be addressed in the EIS. The fourth case was the Darlington Upgrade, a road project in Adelaide, SA. In contrast to NSW, transport infrastructure in SA is legally exempt from the EA process. The project was, however, the subject of an environmental report. This report was undertaken using an EA approach, and supported by a technical template developed by the proponent. The balance of three roads and one light rail project is an approximate reflection of the infrastructure planning space, where there are many more road projects than public transport projects.

### Data Collection

Documents were obtained from government websites (http://majorprojects.planning.nsw.gov.au/ and http://www.infrastructure.sa.gov.au/nsc). As well as EISs or equivalent we also included the requirements issued to each development’s proponents that outline the matters the EIS needs to address. Requirements for the South Australian case were not available. Instead, ‘technical guidelines’ (http://www.dpti.sa.gov.au/standards/environment) were developed by the proponent, the (then named) SA Department for Transport, Energy and Infrastructure.

### Data Analysis

#### Documentary Analysis

A comprehensive documentary analysis coding framework for assessing the inclusion of health in EISs was applied using NVivo software (see Appendix 1). The framework was developed inductively and deductively. It combined previous work by the research team to analyse the content of EISs for health issues^12^ and others who have distilled the essential requirements for including health in impact assessments,^28^ and unpublished work by the World Health Organization (WHO) to assess best practice for considering health in EIAs.^29^ A framework to interrogate policy documents for their inclusion of the social determinants of health^30^ was also used to inform the documentary analysis coding.

The documentary analysis framework comprised six steps designed to capture the ways health was both explicitly and implicitly included within the EISs. First, we coded case attributes, for example location (NSW or SA) and project type (road, light rail). Second, we coded each document for the explicit use of the term ‘health,’ creating a node called ‘health explicit.’ We further coded these instances according to the way the term was used. ‘Health facilities,’ ‘health effects’ and ‘health services’ were examples. We also coded for the term ‘wellbeing.’ The third step was to code for ‘additional detail.’ This included the objectives of the projects, the topics of the EISs, sub-issues, mitigation strategies offered, as well as coding the term ‘equity’ as a strategy or an outcome. In step four we coded the methods that were used for each of the sections of the reports, for example cost benefit analysis or risk assessment. We did this not only for the health section but also for other ‘key issues’ that were assessed such as economic impacts, biodiversity and heritage. In step five, we coded for proposed mitigation measures for each of the ‘key issues’ described by the reports. The sixth step coded for best practice approaches to the technical inclusion of health in EA; whether ‘community health profiles,’ ‘causal pathways,’ ‘use of health data and evidence’ and ‘health equity’ assessments were included or discussed.

## Results

When compared against our framework our analysis showed the EISs partially included health, and that the depth and quality of inclusion varied (findings summarised in Table 2). Within the table, the term ‘insufficient’ has been used to describe when certain elements were present or mentioned, but when compared to best practise were lacking in detail.

**Table 2:** Comparison of EISs From Documentary Analysis

	**NorthConnex**	**CSELR**	**WestConnex M4 East**	**Darlington**
Health part of requirements for EIS	Yes	Yes	Yes	N/A
Consultation with NSW Health as part of requirements for EIS	Yes	Yes	Yes	N/A
Method used to assess health	Risk assessment	As part of SIA	Risk assessment	Health referred to in regards to risk and health protection
Evidence of a broad understanding of health	Yes	Yes	Yes	Yes
Community health baseline/profile (including the existing distribution of mortality, morbidity and health status of affected communities and vulnerable/sensitive sub-groups)	Insufficient	Insufficient	Insufficient	Insufficient
Discussion of the potential associations and causal pathways associated with the project itself, leading to a possible change in one or more health determinants that are likely to cause a change in one or more health outcomes	Partially. Exposure-response relationship noted but focused on specific environmental triggers	Partially. Only in terms of health benefits	Partially. Exposure-response relationship noted across a range of environmental triggers, but emphasizing air quality and not fully developing causal pathways for social impact	No
Health data and evidence: Use of health impact research evidence (qualitative and quantitative)	Yes	Insufficient	Yes	No
Discussion of the possible interactions between project aspects, health determinants, health outcomes and health equity	Insufficient	Insufficient	Insufficient	No
Discussion of the distribution of health impacts across vulnerable/sensitive groups eg, lower socio-economic groups, women, and children	Insufficient	Insufficient	Insufficient	Insufficient

Abbreviations: SIA, social impact assessment; NSW, New South Wales; EIS, environmental impact statement.

### The Importance and Influence of the Requirements Issued to Proponents

Prior to the assessment, each of the NSW cases was issued a set of mandated requirements by the Department of Planning and Environment (see Table 3). Throughout each EIS, the way health was framed and assessed varied according to the mandated requirements.

**Table 3:** Comparison of Requirements Issued to Proponents

**Case**	**Consideration of Health Explicitly Required**	**Section Requiring Consideration of Health**	**Additional Relevant Information**
NorthConnex	Yes	Air Quality: “Consideration of the requirements of Environmental Health Risk Assessment: Guidelines for assessing human health risks from environmental hazards (enHealth, 2012).” Soil and Water: “The assessment of water quality impacts is to have reference to relevant public health and environmental water quality criteria, including those specified in the Australian and New Zealand Guidelines for Fresh and Marine Water Quality (ANZECC/ARMCANZ 2000), and any applicable regional, local or site-specific guidelines.”	Consultation with NSW Health was included in the requirements
CSELR	Yes Health was to be considered as it pertains to land contamination.	Soils, Water and Waste: “land contamination and identification of the need for remediation of contaminated land, having regard to the ecological and human health risks posed by the contamination in the context of past, existing and future land used. Where remediation of contaminated land is required, presentation of a Remediation Strategy taking into account relavent OEH (EPA) guidelines.”	Consultation with NSW Health was included in the requirements
WestConnex M4 East	Yes	Air Quality: “Human health with consideration of: (*a*) how the design of the proposal minimises adverse health impacts, (*b*) human health impacts from the operation of the tunnel under a range of conditions, (*c*) human health risks and costs associated with the proposal associated with air quality, noise and vibration, and social impacts, during the construction and operation of the proposal.”	Consultation with NSW Health was included in the requirements
Darlington	No	No requirements were issued.	Technical guidance was used that did not refer to health

Abbreviations: NSW, New South Wales; OEH (EPA), Office of Environmental Health (Environmental Protection agency).

In their mandatory consideration of health issues, the two NSW road project EISs predominantly focused on risks associated with air quality and noise and vibration, with much less detail on other aspects of health. Both EISs, however, provided a broad definition of the term ‘health,’ acknowledging that it is more than simply an absence of disease. The WestConnex M4 East EIS assessed health in a broader way than the NorthConnex EIS. This was done under the heading ‘Assessment of social impacts on health’ which considered issues such as traffic, public transport, pedestrian and cycle access. In this section there was acknowledgement that the construction phase of the project had the potential to increase stress in the community. There was also a sub-heading within the social impacts on health section titled ‘changes in community’ which discussed issues such as property acquisition and visual changes in the environment. This section was notably brief compared to the assessments of air and noise on health. However, this type of analysis was not explicitly done in any of the other EISs.

The light rail EIS showed that it is possible to include human health in a manner not articulated in the formal requirements. This demonstrated, unlike the road EISs, that health can be considered as both a benefit and a risk of a major transport infrastructure project, and considered as part of a social (and to a lesser extent economic) impact assessment. However, the quality of the analysis of health issues could have been improved (see next section). The limited inclusion of health issues in the 2010 Darlington Upgrade EIS corresponded to the absence of health in the template developed by the proponents.

Mental health was not mentioned in any of the formal assessment requirements issued to proponents. As a result, detailed and comprehensive assessments of the mental health impacts of the construction and operation phases of the projects were absent from all four EISs. When mental health was referred to, it was limited in scope and detail compared to other issues assessed, such as air and noise.

In the NorthConnex EIS, the method used to assess health was human health risk assessment (commonly abbreviated to HHRA). Chapters assessing other issues (such as surface water, biodiversity and heritage) used several methods, including cost benefit analysis, stakeholder analysis, field surveys and inspections. Additionally, the HHRA was based on secondary analysis of the modelling and data presented in other chapters such as the air quality chapter. Within the health risk assessment paradigm, and when assessed against our content analysis framework, the analysis is of high quality: the HHRA used recommended guidance materials and the assessment included additional empirical research, reviews and guidance, detailed community profile data and collection of baseline health data. Equity was considered under the term ‘sensitive receivers,’ focussing on locations where ‘more sensitive’ members of the population such as infants, older people or those with existing health conditions may spend a significant amount of time. The HHRA acknowledges several important caveats and uncertainties within the data. A notable exclusion from the HHRA was ‘Occupational Health & Safety.’ However there are legislative requirements to assess this separately. Asbestos, for example, was assessed separate to the HHRA under ‘workplace hazards.’

The WestConnex M4 East EIS also assessed health using HHRA. This was very similar to NorthConnex, covering all of the previously mentioned elements with the addition of an assessment of social issues within the health section. This is the only case to have explicitly assessed health and social issues together. Equity and differential distribution across population characteristics were included, for example using socio-economic status as a variable for assessing exposure to air quality in the HHRA. This was done within an ‘assessment of social impacts on health’ section, acknowledging that ‘changes within a community that may be associated with the project may be differentially distributed’ and listed various population characteristics such as age, gender and culture. The detail of analysis for health and social issues was, however, not as comprehensive as the detail in the air or noise sections.

The CSELR project principally considered health within its social impact assessment (SIA). There was some consideration in the economic impact assessment appendix but this did not make it into the body of the EIS. Most health references were to health facilities and environmental health. Positive impacts for health were reported such as new opportunities for active transport and so increased physical activity and, subsequently, health and wellbeing. The SIA was done according to cited SIA guidance. Compared with our best practice criteria (as developed in step 6 of the framework), however, the SIA did not include health specific data (although the economic assessment appendix did reference some health data) and did not fully explore causal pathways to specific health outcomes. Equity was considered throughout the EIS under the term ‘sensitive receivers’ defined explicitly in terms of population characteristics (eg, age, gender) and sensitivity to impacts.

The SA case had a very limited assessment of health, only in terms of environmental pollutants in accordance with Australian and state legislation, standards, and guidelines for assessing and managing environmental pollutants (eg, dust, odour, noise, vibration). No social factors were considered. This meant that we could not analyse the report against the best practice categories for including health in EA. Health in the Darlington Transport Study environmental report was discussed in terms of risk and protection from environmental pollutants. In the air quality assessment, health was noted as the principal ‘effect,’ but ‘outcomes’ were not detailed in the same way as they were in the NSW road project EISs. Measures to minimise any ‘nuisance’ or detrimental health effects from dust during the construction phase were proposed in the context of ‘good management practices.’

### Data and Modelling in the Environmental Impact Assessments

The health assessment used secondary data that was taken from the data and modeling presented in other chapters of the EISs (and within the SIA chapter in the light rail). In all cases, regional data was used in place of local health baseline data. Extrapolating from larger populations to locally impacted communities was noted in the EISs as being problematic.

### Reporting of the Development of Mitigation Measures

Regular features of the two road EISs in Sydney were statements alluding to the project having a negligible health risk. The mitigation measures that were offered varied from project to project and according to certain key issues endogenous to the project’s socio-political and biophysical environment. Mitigation measures of impacts to air quality for the road projects were more detailed than those given for noise, vibration and property acquisition.

The process of arriving at mitigation measures was limited or missing. Mitigation methods ranged from actions to be taken by potentially affected individuals, to structural changes to be made by project proponents. An individual level example taken from the WestConnex M4 East EIS was the suggestion that noise and vibration effects of construction will be mitigated by individuals keeping ‘external windows and doors shut and [having] minimal use of outdoor areas.’ Structurally, when operating, the project proposes to mitigate noise impacts through construction of noise barriers, and provision of low noise pavement.

Descriptions of mitigation measures, across issues but particularly concerning noise, were often limited in detail with the qualification that they would be developed in further consultation with the community with more detailed designs to follow at an unspecified date. Because no broader health issues were raised in the SA case this meant that no mitigation measures were detailed there.

### Staged Development and a Lack of Health Promoting Infrastructure Within the Design Parameters

The body of evidence on connections between health and active transport suggests the need for multi-modal transport options. More explicitly, this evidence indicates reductions in the use of private vehicles would create several positive impacts on health, including decreasing exposure to particulate matter, increasing physical activity and reducing the risk of injury and mortality from collisions.^31,32^ In the EISs, the argument for public and active transport infrastructure options was noted and supported. However, detail about what was being committed to within the design parameters of each project varied considerably.

In the NorthConnex EIS there was a section detailing the current situation and future public transport improvements, and the ways the project provides an improved environment for public transport locally and regionally. However, this section concluded with the explicit statement that this is beyond the scope of the project itself:

“*These potential public transport improvements do not form part of this project and would be subject to separate planning processes and approvals as appropriate” (*Volume 1A, p.133)*.*^33^

The WestConnex M4 East EIS articulated several commitments to ‘healthy infrastructure’ including cycling, walking and public transport infrastructure that is affected by the immediate boundaries of the project (ie, no new infrastructure will be provided in this phase of the project)^[1]^. The suggestion was made that other government departments are planning this new infrastructure which is therefore beyond the design parameters being assessed within the EIS:

*“Options for improving public transport along Parramatta Road are at a preliminary stage. More detailed work would be undertaken by Roads and Maritime and Transport for NSW to investigate public transport improvements and intersection treatments that could be delivered on Parramatta Road after the opening of the project. These potential public transport improvements do not form part of this project and would be subject to separate planning processes and approvals as appropriate”* [Volume IA, Chapter 5, p55].^34^

The light rail project is a pertinent comparison case. Its EIS not only demonstrated the health benefits of light rail as a transport option, but also committed the project to providing new health promoting infrastructure surrounding the project itself. Similarly the Darlington study committed to providing a high quality cycling and walking environment through on and off road facilities (the most recent 2016 iteration of this project design announced the extension of a rail line to the teaching hospital which is adjacent to the motorway).

## Discussion

This article presents findings from a novel detailed documentary analysis of the inclusion of health issues in major transport infrastructure EISs. First, our analysis finds that, in relation to the known evidence linking public health and transport policy decisions^1^ and international calls for best practice when including health in EIA,^35^ health was only partially considered in the three Sydney cases, and not considered in the Adelaide case. We judged the consideration to be ‘partial’ for several reasons: social aspects were either not considered or under-considered in terms of detail; the data used to inform the health assessments were secondary and taken directly from the modelling done in other chapters (air and noise for example); providing an assessment of established causal pathways from environmental exposures – beyond but including changes to air quality – through to risk factors and then health outcomes did not occur; health predictions were based on regional data because local data were not available. Nevertheless, the approaches to assessing health using HHRA in the NorthConnex and WestConnex M4 East motorway projects and in the SIA for the light rail were conducted according to current best practice for EA and can on that criterion be considered to be of good quality. However, while acknowledging that some of the deficiencies we noted above are beyond the control of those conducting the health assessments, we remain of the view that when judged against the current evidence base relating transport to health and the more comprehensive WHO guide to best practice^29^ on the coverage of health in these EIAs could have been greatly improved.

Second, our findings also point to the important role of setting requirements, under legislation, for proponents to assess health issues in EISs. The consideration of health was most comprehensive in the WestConnex M4 East EIS, which notably was the only EIS required to have a detailed HHRA as an explicit chapter. The third road project, the Darlington Upgrade in SA, did not assess health other than a brief mention of air quality. As previously mentioned, an EIA is not required under legislation in SA, but would be expected to be included if this jurisdiction were operating under “best practice” approaches. Health was not included in the template to guide the environmental report that was the equivalent of an EIS. The light rail EIS included health as part of the SIA, and to a lesser extent the economic impact assessment, although this was not detailed in the requirements issued.

Navigating the way health is framed in EISs has been a perennial problem.^14,36^ For policy and practice, we have adapted and applied an innovative coding framework to consider the quality of health’s inclusion in the EIA process. The documentary analysis approach used here could be used by practitioners and researchers to determine the quality of the inclusion of health in EISs, and through this to identify areas where additional health input might be useful.^7,28^ This framework may also be used by communities affected by major transport infrastructure projects to help understand and scrutinise EISs. Resident action groups, in particular, would be able to utilise the framework to assist with their advocacy work for a healthier environment.

For research, we add supporting evidence to the broader literature that focusses explicitly on the inclusion of health in transport infrastructure project EISs, which is an increasingly important area for cross-sectoral health focused policy collaboration.^37,38^ Where health was required to be assessed for road projects, this was done using health risk assessment methods focussed on (1) environmental triggers, specifically air and noise, and (2) quantifying exposures and outcomes of the project. These road cases correspond to the long line of research suggesting EISs favour quantification of risks associated with environmental triggers.^14-18^ The light rail project is a useful counter instance, where health was also considered broadly as a benefit of the public transport focussed project, and embedded as a crucial component of the SIA. The fact that causal pathways and health data were not fully utilised in this assessment may be related to the fact that the SIA was not required to focus on health, or use health data (again supporting the power of the legislated requirements). Social issues are known to be the ‘poor cousin’ in EIA.^11,20^ Overall, with the exception of the light rail, coverage of social issues was poor when compared to the detail presented about health risks of air pollution and noise. However, the cases analysed here also indicate that broader, less direct, health impacts of a project, such as those associated with shifting social environments, can be considered central to an EIS. WestConnex M4 East, NorthConnex and the light rail each considered the equitable distribution of health impacts (based on either population characteristics or geographic proximity to the project) in their assessments, and WestConnex M4 East extended the risk assessment to include a brief assessment of mental health issues.

Health impact assessments (HIAs) were absent from the EISs. This is noteworthy as HIAs have been recognised as addressing the limitations of EIA.^39-41^ That HIA was absent confirms an historical preference for specific health risk assessment over the more comprehensive approach of HIA in Australia.^24^

Another major finding for policy, practice and research is that when the content of the EISs was assessed against the known evidence linking health and transport, they generally fell short. Furthermore, the EISs did not consider the project in the context of the health benefits of a multi-modal transport system. Whether it be a different or improved route or mode, major transport infrastructure projects generally provide people with new options for the way they travel. Research suggests that contextual changes, such as the introduction of a new motorway, are important opportunities to influence peoples’ travel behaviour.^42,43^ In cities where the main mode of transport is private car use, major transport infrastructure projects represent fruitful opportunities to promote a shift away from the car and towards more health promoting modes of transport. It is the disruption that matters here. The fact the infrastructure is actually a motorway may be relatively insubstantial. For example, the NorthConnex project is intended to remove traffic from congested local roads, making these roads safer and more pleasant for walking and cycling. The deferral of active transport infrastructure to “separate planning processes and approvals,” as has occurred in the two Sydney road cases, dilutes the potential for the project to catalyse change. The failure of an EIS to consider the full gamut of risks and impacts of the transport system supports our initial supposition that EISs, despite being a regulated mechanism for giving broad consideration to a range of impacts of major infrastructure projects, are not being utilised to reduce the risks and maximise the benefits.

This leads to other important policy questions: whether EIAs are the right point in the life-cycle of an infrastructure project to consider these established connections. The broader literature on transport and health^1^ situates policy recommendations at a higher level, in integrated policy and planning, rather than at an individual project level. The introductory chapters for each EIS covered the broader policy context. These sections articulated higher level policy decisions influencing the project but were silent on health issues. This indicates that health was not considered in the earlier stages of planning these projects. Similarly, the development of options and business cases, and even the design and construction tenders, are crucial but seemingly lost opportunities to introduce health benefit and cost arguments. Methods such as sustainability appraisal (SA) and strategic environmental assessment (SEA) are also opportunities that were not taken up, that may have been useful for including health at an earlier stage. Aside from reference to health ‘benefits’ in the cost benefit analysis of the light rail, these considerations were not mentioned as being included in these earlier decisions. There were, however, regular references to liveability and connectivity in the policy documents mentioned. These issues may be useful entry points for more detailed health arguments and evidence.

There are some limitations to the analysis. First, the findings from four cases cannot be generalised, particularly not to middle and low income countries, non-urban, contexts.^44^ Generalisation was not the primary intent of this research, and we suggest that future research utilises our approach to assess other instances of EIA practice to develop a more comparative picture. Second, although EISs are recognised as the documented output of the EIA process our focus on the content on the EIS may not fully capture the broader process of deliberation that an EIA represents.^6^ Third, we have not separated the construction and operation phases of the projects in the analysis, rather we have assessed how health was considered overall. Further, we are well aware that the content of documents alone cannot explain the wider institutional conditions that influence that content. Unpacking these conditions is the focus of ongoing research by the project team.

## Conclusion

We found that EA is not being fully utilised to promote and protect human health in the major transport infrastructure projects we analysed. The importance of the legislative requirements issued to proponents by the relevant government planning agency is central to this, and a requirement that health be included is necessary to help ensure that health issues are comprehensively considered. While the current focus on human health risks is necessary and, encouragingly, well developed and presented in each of the NSW road EISs, there are limitations to this. The emphasis on risks posed by environmental triggers in EIA is known to be necessary but not sufficient.^23^ This is particularly pertinent to transport focused EISs given the known evidence about the complexity of transport infrastructure’s impact on human health. Major transport projects disrupt local amenity and have costs and benefits within a social determinants of health frame. There are also important policy decisions, particularly around the costs and benefits of focusing on the private car as the main mode of transport. These decisions are made in the early stages of planning a transport project. It is at these stages, when alternatives are being considered, that the health evidence needs the most attention. EIA, when conducted as a regulated process for complying with policy decisions, is not the right mechanism for engaging with the policy decision-making process. We recommend that a comprehensive framework should include a much improved process for assessing the wider impacts of transport on health that includes a social determinants of health approach using a range of methods, including HIA.

## Acknowledgements

The study was funded by a grant from the Henry Halloran Trust, The University of Sydney, Sydney, NSW, Australia. Patrick Harris was funded for this research by the Australian National Health and Medical Research Council, Canberra, ACT, Australia (APP1090644).

## Ethical issues

Ethics approval was granted by the Human Research Ethics Committee, the University of Sydney, Sydney, NSW, Australia. The ethics approval number is 2015/178.

## Competing interests

The authors declare that they have no competing interests.

## Authors’ contributions

PH designed the research with PS, JK, and FB. ER and PH conducted the analysis and wrote up this article. AL conducted the initial analysis on the Darlington case. PS, JK, FB, and AL reviewed this article and provided additional input.

## Authors’ affiliations

^1^Menzies Centre for Health Policy, Sydney, NSW, Australia. ^2^School of Public Health, and Sydney Medical School, The University of Sydney, Sydney, NSW, Australia. ^3^Urban and Regional Planning, Faculty of Architecture, Design, and Planning, The University of Sydney, Sydney, NSW, Australia. ^4^Population Health, South Western Sydney Local Health District, Sydney, NSW, Australia. ^5^Southgate Institute for Health, Society, and Equity, Flinders University, Bedford Park, SA, Australia.

## Endnote

[1] The project approval conditions for WestConnex M4 East confirm that only existing infrastructure impacted upon by the project will be upgraded (http://www.planning.nsw.gov.au/News/2016/Westconnex-M4-East-project-approved).

## Key messages

**Implications for policy makers**

From our research, policy-makers will be able to better:

- Understand the opportunities and limitations of environmental impact assessments (EIAs) as regulated mechanisms for influencing transport infrastructure project decisions.
- Assess the quality of the content of EIAs across a range of types of infrastructure projects from a comprehensive public health impact perspective.
- Critique the setting of requirements for and/or the scoping stage of an EIA to comprehensively include health issues.

**Implications for public**

Transport infrastructure projects are built all around us. environmental impact assessments (EIAs) are regulated opportunities for health advocates and communities to have a say about the potential health impacts of those projects. This research shows how health impacts were considered in four instances of transport infrastructure EIAs: primarily in terms of the health risks arising from air and noise associated with road projects, and as a social benefit in the light rail. Other health issues such as mental health from property acquisitions were not considered in detail. We conclude that EIA currently focusses on environmental risks to health, but we recommend that the process accommodate a more comprehensive and holistic understanding of health and its social determinants and the influence of transport on these.

## Appendix 1

### Appendix 1. Coding Framework for Investigating the Coverage of Health in EISs

**Table:**

**Step**	**Focus**
Step 1: Attribute coding	Attributes of the case found in the EIS and requirements (eg, tunnel, urban)
Step 2: ‘Health’ explicit	‘Health’ and ‘wellbeing’ and derivatives of these which mention either word ‘health’ or wellbeing sections/chapters
Step 3: Additional detail	• **Objectives** (for the project being assessed/for the EIA) • **Topics** (in the EIAs *against chapters, headings*) • **Sub-issues/impacts/outcomes** – eg, air quality, noise, social wellbeing • Health outcomes (mental health, mortality) Environmental outcomes (reduced pollution, reduced noise, traffic flow) • Social outcomes (wellbeing, isolation) • Economic outcomes (business, jobs) • Behavioural outcomes (increased physical activity, reduced behavioural risk factors) • **Strategies** • Structural eg, road pricing, active travel, multi-modal transport • Individual behaviour eg, active travel, car sharing • **Equity** (strategy or outcome) - Spatial, temporal, socio-demographic groups
Step 4: Method used In the health assessment In assessments of other issues	• Primary data collection – qualitative, quantitative and how? • Stakeholder analysis • Baseline • Secondary data (statistics, documents, literature) • Risk assessment – quantitative and qualitative • Cost benefit analysis • Modelling • Other
Step 5: Mitigation measures • In the health assessment • In assessments of other issues	What mitigation measures are proposed?
Step 6: Best practice approach to technical inclusion of health in EIA Including and assessment of the quality of the information used	1. **Community health baseline/profile:** (including the existing distribution of mortality, morbidity and health status of affected communities and vulnerable/sensitive sub-groups) 2. **Causal pathways:** [Evidence-informed?] discussion of the potential associations and causal pathways from a ‘project aspect’ (project process or activity) leading to a possible change in one or more health determinants that are likely to cause a change in one or more health outcomes (eg, communicable disease, non-communicable disease) 3. **Health data and evidence:** Use of health impact research evidence, qualitative and quantitative, to identify causal pathways and the significance of a health impact 4. **Health equity:** Discussion of the possible interactions between project aspects, health determinants, health outcomes and health equity. Discussion of the distribution of health impacts across vulnerable/sensitive groups eg, lower socio-economic groups, women, children
Step 7: Discourse analysis questions	1. **Regulatory**, strategic and business case context - under what regulatory, strategic policy and business case conditions will the project be assessed? 2. **Social relations** – who did what and for whom? 3. **Language** – what was being represented and the clarity of assumptions, analysis, conclusions? 4. **Pre-suppositions** – what policy and other assumptions drove the content of the EIS? 5. **Intent/purpose** – what was being committed to in the EIS?

Abbreviations: EIA, environmental impact assessment; EIS, environmental impact statement.
